# Evolution of siRNA Therapeutics: From Mechanistic Foundations to Clinical Expansion

**DOI:** 10.3390/pharmaceutics18050593

**Published:** 2026-05-12

**Authors:** Quoc-Viet Le, Gayong Shim

**Affiliations:** 1 Research Group in Pharmaceutical and Biomedical Sciences, Faculty of Pharmacy, Ton Duc Thang University, Ho Chi Minh City 72912, Vietnam; lequocviet@tdtu.edu.vn; 2 School of Systems Biomedical Science, Soongsil University, Seoul 06978, Republic of Korea; 3 AI-Bio Convergence Research Institute, Soongsil University, Seoul 06978, Republic of Korea

**Keywords:** RNA interference, small interfering RNA, GalNAc-siRNA conjugates, chemical modification of oligonucleotides

## Abstract

Since the discovery of RNA interference (RNAi), small interfering RNA (siRNA) has emerged as a transformative therapeutic modality, shifting the paradigm from permanent genomic modification to the flexible interception of genetic information. Despite the delivery gap caused by biological barriers, innovations in chemical stabilization and delivery platforms have propelled siRNA from niche applications to the mainstream management of chronic conditions. This review provides a comprehensive analysis of the distinct mechanistic advantages of siRNA over antisense oligonucleotides, with particular emphasis on its catalytic turnover via the RISC and high target specificity. We further evaluate the critical transition from first-generation lipid nanoparticles to ligand-conjugated systems, specifically trivalent N-acetylgalactosamine (GalNAc). Through an examination of the clinical success of Inclisiran and the recent approval of Plozasiran, we discuss how these advances have improved patient compliance and extended dosing intervals. Furthermore, this article explores the emerging frontier of extra-hepatic delivery and the expansion toward metabolic and oncological targets. Ultimately, this review highlights the potential of siRNA to become a programmable standard of care for a broad spectrum of previously intractable diseases.

## 1. Introduction

Since the initial conceptualization of gene therapy in the 1970s, the utilization of biological genetic information for disease treatment has undergone remarkable evolution [1]. While traditional gene therapy primarily focuses on the DNA level—utilizing viral vectors within the nucleus to achieve gene augmentation or editing for long-term and often irreversible phenotypic changes—the emergence of nucleic acid therapeutics, particularly those based on small interfering RNA (siRNA), has established a distinct therapeutic paradigm [2,3,4,5,6,7]. Unlike DNA-based approaches, siRNA operates within the cytoplasm, targeting mature messenger RNA (mRNA) to induce selective gene silencing [8]. This non-integrative and transient mode of action fundamentally eliminates the risks associated with genomic alterations and enables precise pharmacokinetic control through dose titration, offering substantial advantages in terms of both safety and regulatory compliance over conventional gene therapies. Consequently, if traditional gene therapy is defined by the permanent modification of the genetic blueprint, siRNA-based therapy represents the flexible interception of transmitted information. This shift allows for the modulation of undruggable disease-related proteins that have remained inaccessible to conventional small molecules or monoclonal antibodies, thereby providing a transformative solution for previously intractable conditions [9].

These distinctions provide the fundamental rationale for the superior pharmacokinetic safety and regulatory advantages of RNA therapeutics [10,11]. The non-integrative nature, which eliminates the risk of insertional mutagenesis, combined with a transient mode of action that allows for reversible dose titration, effectively addresses the long-standing challenges of unpredictable genetic alterations and long-term toxicity often associated with traditional gene therapies. Consequently, these attributes offer a more predictable safety profile during clinical development and regulatory review. This robustness has established a technical foundation for expanding siRNA applications beyond rare genetic disorders toward more prevalent chronic conditions where long-term safety and titratability are paramount.

Despite this theoretical potential, a significant delivery gap persisted for two decades between the discovery of the RNA interference (RNAi) mechanism in 1998 and the approval of the first siRNA drug [12,13]. This technological hiatus was primarily due to the formidable biological barriers encountered upon systemic administration. Exogenous siRNAs are highly susceptible to rapid degradation by serum nucleases, resulting in an extremely short circulatory half-life. Furthermore, their relatively high molecular weight and dense polyanionic charge hinder spontaneous translocation across the hydrophobic cellular membrane. The propensity of the innate immune system to recognize these exogenous sequences as viral mimics, thereby triggering potent immunogenic responses, further complicated early clinical translation [8].

Ultimately, the history of siRNA therapeutics has been defined by the strategic quest to overcome these delivery challenges. The maturation of the field was made possible through synergistic breakthroughs in chemical modification and the development of sophisticated delivery platforms, notably lipid nanoparticles (LNPs) and GalNAc conjugates [14,15]. While the FDA approval of Onpattro (patisiran) marked a triumphant milestone as the first LNP-based siRNA drug, the subsequent clinical success and expansion of the field have been largely driven by the advance of ligand-conjugate technologies. Together, these innovations effectively bridged the delivery gap, signaling the dawn of a new era in molecular medicine where genetic information is utilized as a programmable therapeutic tool to address a broad spectrum of diseases.

This review aims to delineate the mechanistic foundations of siRNA, evaluate the evolution of delivery systems from LNPs to next-generation GalNAc platforms, and discuss how these innovations are bridging the gap between rare genetic disorders and high-prevalence chronic diseases, ultimately signaling a new era of programmable molecular medicine.

## 2. Mechanisms of RNA Interference

### 2.1. Mechanistic Comparison: siRNA vs. ASO

While both siRNA and antisense oligonucleotides (ASOs) aim to modulate gene expression, they function through fundamentally different biological pathways. ASOs are typically single-stranded DNA analogs that bind to target mRNA to form a DNA/RNA heteroduplex [16,17]. This hybrid structure recruits RNase H1, a ubiquitous endogenous enzyme that subsequently cleaves the RNA strand in a 1:1 stoichiometric manner. This occupancy-based mechanism requires a constant high concentration of ASOs to maintain silencing. In contrast, siRNA is introduced as a double-stranded RNA molecule that is incorporated into the RNA-induced silencing complex (RISC) [18]. By harnessing this specialized multi-protein machinery, siRNA achieves a sophisticated enzymatic and catalytic silencing effect. This allows for significantly higher potency at much lower concentrations (picomolar range) compared to the stoichiometric turnover of ASOs [19].

### 2.2. Evolutionary Origins and Endogenous siRNA

RNA interference is an evolutionarily conserved intracellular defense system originally developed to safeguard genomic integrity against exogenous viral threats and the mobilization of retrotransposons or pseudogenes [20]. In mammalian systems, particularly in oocytes and embryonic stem cells, endogenous siRNAs (endo-siRNAs) are generated from long double-stranded RNA transcripts through Dicer-mediated processing (an RNase III-like enzyme) [21,22]. These endo-siRNAs play a critical role in maintaining heterochromatin stability and regulating transcript levels [23]. Modern siRNA therapeutics are designed to hijack this innate immune-like machinery by introducing exogenous sequences into the pre-existing RISC pathway. By utilizing this pre-established endogenous biological program, siRNA drugs ensure high biocompatibility and exceptional silencing efficiency, as they work within the cell’s natural regulatory framework.

### 2.3. Catalytic Turnover of the RISC

The primary driver of sustained therapeutic efficacy of siRNA is the catalytic turnover facilitated by the RISC. Once the guide strand is loaded onto Argonaute 2, the catalytic core of RISC possessing a PIWI domain with RNase H-like activity, it directs the site-specific cleavage (slicing) of the target mRNA [24,25]. Crucially, the guide strand is not consumed or degraded during this process; instead, it remains protected within the Argonaute 2 pocket. This allows the complex to release the cleaved mRNA fragments and proceed to recognize and destroy subsequent target molecules [26,27]. This repetitive enzymatic cycle (multiple turnover) enables a single siRNA molecule to silence hundreds of mRNA copies, resulting in a prolonged duration of action (often lasting weeks) and a significantly reduced dosing requirement, which in turn minimizes the potential for off-target toxicities and systemic side effects.

### 2.4. Functional Divergence from miRNA: Ensuring Target Specificity

Although siRNA and microRNA (miRNA) share the RISC-mediated pathway, they are distinguished by their degree of target complementarity and their subsequent mode of action [28,29]. Endogenous miRNAs typically exhibit partial complementarity to their target mRNAs, primarily through a short seed region (nucleotides 2–8) at the 3′ UTR, which leads to translational repression and the broad regulation of hundreds of genes simultaneously [30,31]. Mechanistically, miRNA-loaded RISC typically prevents protein synthesis by interfering with the assembly of the translation initiation complex to induce mRNA deadenylation and decapping. This allows for the effective silencing of gene expression through protein synthesis inhibition, often preceding or occurring alongside mRNA decay, without the requirement for direct transcript cleavage. Conversely, therapeutic siRNAs are engineered for full, high-fidelity complementarity with their target sequences [32,33]. This perfect base-pairing induces direct endonucleolytic cleavage mediated by the Argonaute-2 protein at a specific site, rather than broad suppression or sequestration in P-bodies. This one-to-one precision is the hallmark of siRNA technology, fulfilling the requirements of precision medicine by ensuring the highly selective silencing of a single disease-causing gene while mitigating pleiotropic off-target effects that often plague broader-acting oligonucleotides [34]. While miRNAs act as rheostats for entire networks of genes, siRNAs function as a surgical tool for the absolute knockdown of a specific transcript.

## 3. Technologies for siRNA-Based Therapeutics

The clinical success of siRNA-based therapeutics is predicated on sophisticated chemical modifications and robust delivery systems designed to circumvent rapid enzymatic degradation and innate immune recognition. The evolution of these technologies has significantly expanded the therapeutic index and prolonged the duration of action for modern RNAi drugs. In particular, recently approved products have expanded their scope from rare genetic diseases to chronic conditions such as hyperlipidemia.

### 3.1. Chemical Modifications for Stability and Therapeutic Index

The transition of siRNA from a laboratory tool to a clinical reality was made possible by precise modifications to the phosphate backbone, ribose sugar, and termini. These alterations are summarized in Table 1. Recent design strategies, such as Alnylam’s ESC (Enhanced Stabilization Chemistry) and ESC+ platforms, utilize an optimized alternating pattern of 2′-OMe and 2′-F modifications to ensure double-strand stability and reduce immunogenicity [35,36]. The ESC platform, combined with GalNAc conjugation, has enabled prolonged efficacy with dosing intervals of up to six months, as famously seen with Inclisiran. Building on this, the ESC+ platform incorporates specialized moieties like glycol nucleic acid (GNA) at specific positions on the antisense strand to minimize seed-mediated off-target effects, a strategy successfully applied in newer clinical therapeutics like Elebsiran [37,38,39,40,41]. With the exception of the LNP-encapsulated Onpattro (Patisiran), all currently approved siRNA drugs are conjugates that utilize phosphorothioate (PS) linkages, which replace a non-bridging oxygen with sulfur to significantly enhance resistance against nuclease degradation [42].

The transition from labile phosphodiester-linked RNAs to clinical therapeutics necessitates a profound understanding of the molecular interactions between the siRNA and its biological environment. The primary rationale for 2′-ribose modifications, such as 2′-O-methyl (2′-OMe) and 2′-fluoro (2′-F), is twofold: to sterically hinder nuclease access and to modulate the thermodynamic stability of the siRNA duplex [43,44]. 2′-OMe modifications mimic the natural ribose structure but eliminate the 2′-hydroxyl group, a common site for nucleophilic attack during RNA hydrolysis. In contrast, the high electronegativity of fluorine in 2′-F modifications induces a C3′-endo sugar pucker, which pre-organizes the RNA strand into a stable A-form helix, thereby enhancing the binding affinity to the target mRNA [45].

Furthermore, the placement of PS linkages at the terminal ends of the duplex provides essential protection against exonucleases. However, since excessive PS substitution can lead to non-specific protein binding and subsequent cytotoxicity, modern gapmer or alternating designs utilize a minimal PS footprint [46]. Recent innovations, such as the incorporation of GNA or S-GNA, further refine this stability by introducing thermal destabilization at specific points [47,48]. This destabilization is paradoxically beneficial; it reduces seed-region-mediated off-target effects by decreasing the binding energy of the siRNA guide strand to non-target transcripts, thereby sharpening the therapeutic window.

**Table 1:** Chemical Modifications in Major siRNA Platforms.

Category	Modification	Mechanism and Function	Major Platforms	Reference
Backbone	Phosphorothioate	Replaces non-bridging oxygen with sulfur to resist exonuclease degradation.	STC, ESC, ESC+	[6,16,46]
Sugar	2′-O-Methyl	Eliminates the 2′-hydroxyl group to block immunogenicity and enhance metabolic stability.	STC, ESC, ESC+	[6,13,16,45]
	2′-Fluoro	Induces a C3′-endo sugar pucker to maximize binding affinity for target mRNA.	STC, ESC, ESC+	[6,16,43,45]
Ring/ Acyclic	(S)-glycol nucleic acid	Induces localized thermal destabilization in the seed region to reduce off-target effects.	ESC+	[15,35,41,48]
Terminus	5′(E)-vinyl phosphonate	Mimics the 5′-phosphate to resist phosphatase cleavage and anchor the guide strand into Ago2.	C16-siRNA platform	[16,49,50]
Structural architecture	Tetraloop	Stabilizes the duplex and provides an extended loop region for multivalent GalNAc conjugation.	GalXC™	[13,36]
Targeting	Trivalent GalNAc	Enables highly efficient ASGPR-mediated targeted delivery to hepatocytes.	SC, ESC+, GalXC™	[38,51,52,53]
	Lipid conjugate (C16)	Facilitates extra-hepatic delivery (e.g., CNS) by enhancing lipophilicity and broad cellular uptake.	C16-siRNA platform	[49,50,54]

ASGPR, asialoglycoprotein receptor; C16, hexadecanoic acid (palmitic acid) conjugate; ESC/ESC+, enhanced stabilization chemistry; GalNAc, N-acetylgalactosamine; STC, standard template chemistry.

### 3.2. Advanced Delivery Platforms: From LNPs to Ligand Conjugates

Because siRNA is a large, negatively charged molecule, it cannot spontaneously cross the hydrophobic cellular membrane. Therefore, specialized delivery systems are required to facilitate its transport into the cytoplasm [55].

#### 3.2.1. Lipid Nanoparticles (LNPs)

As the platform behind the first FDA-approved siRNA drug, Onpattro, LNPs encapsulate siRNA within a four-component system: ionizable cationic lipids, helper lipids, cholesterol, and PEG-lipids [56]. The pivotal feature of the LNP is its ability to facilitate endosomal escape. In the acidic environment of the endosome, the ionizable lipids (e.g., DLin-MC3-DMA) become protonated (positively charged), triggering a fusion event with the anionic endosomal membrane that releases the siRNA into the cytosol [57]. Onpattro (Patisiran) specifically utilizes this LNP delivery method and requires intravenous (IV) infusion every three weeks. Importantly, the optimization of LNP formulation and delivery was crucial for the rapid enablement and unprecedented success of mRNA vaccines for COVID-19. The extensive foundational work with Onpattro, along with other early developments in the RNAi field, established the manufacturing, safety, and regulatory precedents that paved the way for these historic vaccines [56,58].

#### 3.2.2. Trivalent GalNAc Conjugates

The development of GalNAc conjugates represented a paradigm shift in siRNA delivery [15]. This technology involves the direct conjugation of N-acetylgalactosamine to the siRNA sense strand, typically at the 3′terminus [51]. Specifically, the trivalent configuration of GalNAc molecules forms a high-avidity bond with the asialoglycoprotein receptor (ASGPR), which is abundantly and specifically expressed on hepatocytes. This ligand-mediated approach allows for subcutaneous administration, dramatically improving patient compliance and convenience compared to intravenous infusions. Starting with Givlaari (approved 2019), subsequent approvals such as Oxlumo, Leqvio, and Amvuttra have all utilized GalNAc-mediated delivery, capitalizing on this subcutaneous route to successfully extend dosing intervals from monthly to as long as every six months. Most recently, Plozasiran (approved 2025) has continued this trend by utilizing the GalNAc-based TRIM platform to facilitate convenient subcutaneous self-injection. Building upon these successes, the evolution of conjugate chemistry has led to Alnylam’s next-generation IKARIA™ platform [59]. Specifically engineered for ultra-long durability, the IKARIA platform further optimizes chemical modification patterns to maximize metabolic stability and RISC loading efficiency, aiming to extend dosing intervals toward once-yearly administration.

### 3.3. Manufacturing and Quality Control: Ensuring Scalability

As siRNA therapeutics move toward large-scale chronic disease markets, the chemistry, manufacturing, and controls (CMC) aspect becomes a critical determinant of commercial success [60]. Regardless of the delivery platform, the production of all siRNA therapeutics begins with the automated solid-phase synthesis of the oligonucleotide strands using phosphoramidite chemistry. This foundational step ensures high-purity drug substances before any subsequent conjugation or formulation occurs [60].

For GalNAc conjugates, the manufacturing process has become highly streamlined and efficient [60,61]. The trivalent GalNAc cluster is typically integrated during the solid-phase synthesis or attached via well-characterized conjugation chemistry, allowing for high molecular homogeneity. While maintaining purity and monitoring process-related impurities remain essential, the industrial-scale production of GalNAc-siRNA is now a robust and reproducible process that supports the commercial viability of multi-kilogram batches.

In contrast, LNP-encapsulated siRNAs, such as Patisiran, involve a distinct two-stage manufacturing workflow: the synthesis and purification of the siRNA drug substance, followed by its formulation into lipid nanoparticles. This second stage requires sophisticated microfluidic mixing technology [58]. To achieve a uniform polydispersity index, the lipid components (in ethanol) and the siRNA (in an acidic aqueous buffer) must collide at precise flow rates and ratios. This rapid mixing triggers the self-assembly of the nanoparticles, trapping the siRNA within the hydrophobic core. Quality control for LNPs is exceptionally stringent, focusing on particle size distribution, encapsulation efficiency (typically >90%), and the stability of the ionizable lipid components. As the field matures, the development of “one-pot” synthesis methods and continuous manufacturing processes is being explored to reduce production costs and facilitate the global distribution of siRNA therapeutics.

## 4. FDA-Approved siRNA Therapeutics: Transition from Rare to Chronic Diseases

Since the landmark approval of the first siRNA therapeutic in 2018, the landscape of RNAi has rapidly evolved. Initially focused on ultra-rare genetic disorders, siRNA technology is now penetrating large-scale chronic disease markets. This progression represents a significant paradigm shift in patient-centric care, characterized by the transition from intravenous to subcutaneous administration and a remarkable extension of dosing intervals. The evolution from LNP-based delivery to GalNAc conjugation has been the primary driver behind this increased clinical utility and patient convenience. Table 2 provides a summary of these FDA-approved siRNA therapeutics, detailing their molecular targets, delivery platforms, and evolving clinical indications.

### 4.1. The First-Generation LNP Platform: Patisiran (Onpattro)

Patisiran, approved by the FDA in 2018 for the treatment of polyneuropathy in hereditary transthyretin-mediated (hATTR) amyloidosis, serves as the foundational proof-of-concept for siRNA-based medicine [62]. Patisiran established the clinical viability of LNP platforms for targeted hepatic delivery [63]. By utilizing RNAi to silence the TTR gene, it addresses the root cause of the disease—the production of amyloidogenic TTR protein—rather than merely managing downstream symptoms. Technically, it utilizes the DLin-MC3-DMA ionizable lipid to facilitate endosomal escape. Despite its success, the first-generation LNP platform faces challenges in patient compliance [62,64]. The requirement for intravenous infusion every three weeks and the necessity of corticosteroid and antihistamine premedication to mitigate infusion-related reactions underscored the need for more refined delivery systems [64].

### 4.2. Innovation via GalNAc Conjugation: Precision and Convenience

The development of GalNAc conjugation technology has been a transformative leap, enabling potent, liver-specific delivery via subcutaneous injection [61]. The GalNAc platform has enabled the precise targeting of various metabolic pathways within the liver. Givosiran (Givlaari) targets ALAS1 mRNA for the treatment of acute hepatic porphyria and was the first GalNAc-siRNA conjugate to receive approval, administered monthly. The approval of Lumasiran (Oxlumo) and Nedosiran (Rivfloza) demonstrates the maturity of the technology, where different enzymes (GO and LDH, respectively) within the same metabolic pathway can be targeted to optimize patient outcomes in primary hyperoxaluria type 1. Vutrisiran (Amvuttra), the successor to Patisiran, utilizes enhanced stabilization chemistry (ESC)-GalNAc technology. This advance allows for subcutaneous administration every three to six months, depending on the indication and target, significantly reducing the treatment burden and improving the quality of life by replacing the three-weekly IV infusions of Patisiran.

### 4.3. Expansion into the Blockbuster Market: Chronic Disease Management

The current trajectory of siRNA therapeutics is marked by their entry into high-prevalence chronic disease sectors, such as cardiovascular and metabolic health [69,70]. Inclisiran (Leqvio) represents a major milestone as an siRNA-based treatment for hypercholesterolemia. By silencing PCSK9 mRNA, it achieves sustained reduction in LDL cholesterol [65]. Its semi-annual (twice-yearly) dosing schedule addresses the chronic issue of medication non-adherence, offering a vaccine-like prophylactic approach to cardiovascular disease management. Notably, Inclisiran utilizes the ESC platform to ensure high metabolic stability, allowing for the extended 6-month dosing interval.

The scope of RNAi has significantly expanded with the recent approvals of breakthrough therapies. Now clinically available, Fitusiran targets antithrombin to rebalance coagulation in hemophilia A and B, providing a potent, long-acting alternative to traditional factor replacement [67]. Its success in clinical trials has translated into a transformative reduction in the annualized bleeding rate for patients. However, its clinical implementation requires stringent monitoring of antithrombin levels to maintain a narrow therapeutic window and mitigate the risk of thrombotic events [71,72]. Significant safety considerations have been identified, including vascular thrombosis, gallbladder-related events (such as cholelithiasis), and transient elevations in hepatic enzymes. Managing these risks through refined dosing regimens is essential for balancing its profound efficacy with patient safety. Similarly, Plozasiran has secured approval for familial chylomicronemia syndrome [68]. With data demonstrating a profound and durable reduction in triglyceride levels, it is firmly establishing siRNA as a standard of care in metabolic medicine.

## 5. Challenges and Future Perspectives

**Figure 1:**
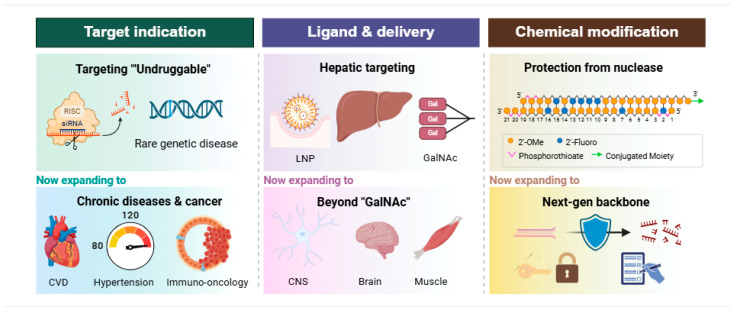
Paradigm shifts in the development of siRNA-based modalities. The development of RNAi technology relies on three interconnected strategies. Disease application is broadening from niche genetic conditions to systemic and malignant pathologies. Concurrently, tropism and vector design are moving past traditional liver-centric accumulation, pioneering precise intracellular delivery into hard-to-deliver extrahepatic microenvironments. Ultimately, nucleic acid chemistry is evolving from basic stability enhancements toward sophisticated structural innovations that maximize the therapeutic index and secure patentability (Created in Biorender. Shim, G. (2026) https://BioRender.com/vt4qymv, accessed on 8 May 2026).

While siRNA therapeutics have achieved dominance in hepatic targets, the next frontier involves overcoming the barriers to extra-hepatic delivery and expanding into complex metabolic and oncological indications [66]. This evolution is driven by three core pillars (Figure 1): selecting targets with clear genetic causes, discovering novel ligands for extra-hepatic delivery, and implementing advanced chemical modifications to ensure stability and reduce immune-related side effects.

### 5.1. Extra-Hepatic Delivery: Overcoming Tissue-Specific Barriers

Recent advances in ligand engineering and chemical modifications are enabling siRNA to reach beyond the liver. Leading companies are developing proprietary platforms to bypass hepatic sequestration. For instance, C16 conjugate technology enhances lipophilicity by appending a C16 (2′-O-hexadecyl) moiety to the siRNA terminus, significantly improving cell membrane penetration and enabling broad distribution throughout the CNS via intrathecal administration [49]. Similarly, the pulmonary TRiM platform utilizes optimized ligands and linkers to target lung tissues (alveolar and airway epithelium) through both inhaled and subcutaneous routes [73]. GalXC-Plus further extends this reach into muscle and adipose tissues by incorporating specific chemical modifications that enhance tissue retention and activity [74]. A particularly significant breakthrough in extra-hepatic targeting is the development of antibody–oligonucleotide conjugates (AOCs), which utilize monoclonal antibodies to exploit cell-surface receptors for tissue-specific uptake [75,76].

The clinical impact of these technologies is already evident. ALN-APP, currently in clinical trials, has demonstrated a sustained reduction in amyloid precursor protein in the cerebrospinal fluid, offering a potential breakthrough for Alzheimer’s disease [50]. For respiratory diseases, inhaled siRNA delivery is gaining traction. ARO-RAGE has shown a potent (up to 90%) silencing of target genes in the lung with minimal systemic exposure, providing a new therapeutic avenue for asthma and idiopathic pulmonary fibrosis [77]. In the realm of neuromuscular diseases, Avidity Biosciences has reported landmark success with its AOC platform. Delpacibart etedesiran (del-desiran, AOC 1001), targeting *DMPK* mRNA for the treatment of Myotonic Dystrophy Type 1 (DM1), demonstrated a ~46% reduction in muscle *DMPK* levels and a significant amelioration of aberrant splicing patterns in a Phase 1–2 trial [75]. Building on this, delpacibart braxlosiran (del-brax, AOC 1020) has shown the potential to treat Facioscapulohumeral Muscular Dystrophy (FSHD) by reducing *DUX4*-regulated gene expression by approximately 75% [76]. These developments mark a pivotal shift in RNAi therapeutics, moving from liver-centric applications to the precise systemic treatment of muscular and neurological disorders.

### 5.2. Strategic Shift: From LNP to GalNAc Conjugates

A major trend following the success of Onpattro is the strategic transition from lipid nanoparticles to GalNAc conjugate platforms (ESC/ESC+). This shift is driven by a combination of intellectual property considerations, patient convenience, and safety profiles. From a business perspective, many companies are seeking patent independence by moving away from foundational LNP patents [78]. By establishing their own proprietary GalNAc platforms, such as LICA (Ligand-conjugated antisense oligonucleotide, Ionis) [79] or TRiM (Targeted RNAi Molecule, Arrowhead) [80], they can reduce heavy royalty burdens and secure long-term technological leadership. This is particularly crucial as first-generation GalNAc patents approach their expiration in the mid-2020s, prompting late-comers to adopt circumvention strategies like modifying the chemical structure of linkers, adjusting the valency of GalNAc clusters, or fine-tuning the sugar moieties to bypass core IP [52].

Beyond the legal landscape, the shift to GalNAc conjugates offers transformative improvements in clinical administration and safety. While LNP-based Onpattro requires complex intravenous infusions every three weeks—often necessitating premedication to mitigate infusion-related reactions—GalNAc conjugates allow for subcutaneous injection with a significantly reduced dosing frequency of every few months [53]. This transition not only enhances patient adherence through self-injection options but also offers a superior safety profile. GalNAc conjugates typically exhibit higher biocompatibility and a smaller molecular footprint compared to LNPs, which are frequently associated with dose-limiting toxicities or immune responses triggered by the lipid components themselves [81]. Consequently, the high-affinity binding of GalNAc to the ASGPR on hepatocytes ensures efficient, targeted delivery with minimal off-target effects, solidifying its position as the current gold standard for hepatic siRNA therapy.

### 5.3. Indication Expansion and Market Trends

The market is witnessing a diversification of GalNAc technologies. Alnylam utilizes trivalent GalNAc clusters attached to the 3′ end of the sense strand [51], while Ionis employs its LICA platform with GalNAc at either the 5′ or 3′ end of ASOs [82]. Dicerna stands out with its Tetraloop structure (GalXC), where GalNAc is directly conjugated to the siRNA loop [83]. As first-generation GalNAc patents begin to expire in the 2020s, late-comers are employing patent circumvention strategies. This includes modifying the chemical structure of linkers, adjusting the valency of GalNAc, or fine-tuning the sugar moieties to build a “technology shield” against foundational IP. In terms of clinical applications, Zilebesiran, targeting angiotensinogen, has entered Phase 3 clinical trials (ZENITH) following the KARDIA Phase 2 program, which demonstrated sustained blood pressure reduction for up to six months with a single dose [84]. Furthermore, siRNA targeting PNPLA3 is being explored as a definitive treatment for metabolic dysfunction-associated steatohepatitis [85]. The siRNA-based strategies are also targeting “undruggable” oncogenes like KRAS [86]. The siG12D-LODER implant has shown promising results in extending overall survival in pancreatic cancer patients by providing localized, sustained release of siRNA directly into the tumor microenvironment.

### 5.4. AI-Driven Design and Predictive Modeling: The Future of Precision RNAi

The integration of artificial intelligence (AI) and machine learning is fundamentally redefining the siRNA drug discovery pipeline [87,88,89]. Traditionally, selecting a potent siRNA sequence involved laborious screening of hundreds of candidates across a target mRNA. However, deep learning algorithms, trained on vast datasets of siRNA silencing efficiency and off-target profiles, can now predict the slicing potency of a sequence with unprecedented accuracy. These models analyze complex features, such as the thermodynamic stability of the siRNA-mRNA duplex, the accessibility of the target secondary structure, and the presence of specific motifs that favor Argonaute 2 loading.

One of the most critical contributions of AI in this field is the mitigation of off-target effects [90]. Advanced bioinformatic tools utilize neural networks to simulate seed-region interactions across the entire human transcriptome. By predicting potential unintended binding events before synthesis, researchers can engineer sequences that maximize on-target silencing while maintaining a clean safety profile. Furthermore, AI is being employed to optimize chemical modification patterns. Given the nearly infinite combinations of 2′-OMe, 2′-F, and PS linkages, generative AI models can identify the optimal “stabilization fingerprint” for a specific tissue, significantly reducing the time and cost of lead optimization.

Beyond sequence design, AI is also accelerating the discovery of novel extra-hepatic delivery ligands [54]. By virtual screening of chemical libraries and modeling the binding affinity between ligands and tissue-specific receptors, AI-driven platforms are bridging the gap for non-liver applications. As the field moves toward programmable medicine, the synergy between high-throughput experimental data and predictive modeling will likely establish a self-evolving loop, where each clinical success further refines the algorithms, ultimately leading to a new generation of highly personalized and potent RNAi therapeutics.

## 6. Conclusions

The evolution of siRNA therapeutics represents one of the most significant leaps in modern pharmacology, successfully transitioning from a laboratory phenomenon to a robust clinical reality. By hijacking the endogenous RISC machinery, siRNA offers a unique combination of catalytic potency and high-fidelity specificity, overcoming the stoichiometric limitations of traditional antisense approaches. The fundamental shift from DNA-based permanent modification to RNA-based transient modulation has provided a safer, titratable, and more predictable regulatory pathway for genetic medicine.

The trajectory of siRNA technology has been defined by the resolution of the delivery challenge. The move from systemic LNP-mediated delivery to targeted GalNAc conjugation has not only minimized off-target toxicities and infusion-related reactions but also transformed the patient experience. The transition from frequent intravenous infusions to semi-annual subcutaneous injections—as seen in the vaccine-like model—addresses the perennial challenge of medication adherence in chronic disease management. Furthermore, the expansion of the therapeutic pipeline into prevalent conditions such as hypertension, dyslipidemia, and even undruggable oncological targets underscores the versatility of RNAi as a programmable tool.

Looking forward, the post-GalNAc era will be characterized by two primary trends: the conquest of extra-hepatic tissues and the navigation of the shifting intellectual property landscape. The development of C16 conjugates for the CNS and TRiM platforms for pulmonary delivery is already yielding promising clinical data, suggesting that the benefits of siRNA will soon extend to neurodegenerative and respiratory diseases. Concurrently, as foundational patents expire, the industry is seeing a surge in chemical diversity, with novel linkers, valency adjustments, and backbone modifications enhancing the therapeutic index even further.

In conclusion, siRNA therapeutics have bridged the gap between genetic potential and clinical application. As delivery platforms continue to achieve higher tissue selectivity and chemical modifications further extend metabolic stability, siRNA is set to establish itself as a cornerstone of precision medicine, providing a scalable and programmable solution for both rare and common diseases alike.

## Figures and Tables

**Table 2:** Chronological overview of FDA-approved siRNA therapeutics.

Drug (Brand Name)	Developer	Molecular Target/ Indication	Delivery Platform	FDA Approval	Reference
Patisiran (Onpattro)	Alnylam	TTR/ hATTR-polyneuropathy	Lipid nanoparticles	2018	[10,12,56,62,63,64]
Givosiran (Givlaari)	Alnylam	ALAS1/ Acute hepatic porphyria	GalNAc-siRNA	2019	[10,38,61]
Lumasiran (Oxlumo)	Alnylam	HAO1/ Primary hyperoxaluria type 1	GalNAc-siRNA	2020	[10,38]
Inclisiran (Leqvio)	Novartis	PCSK9/ Hypercholesterolemia	GalNAc-siRNA	2021	[10,65]
Vutrisiran (Amvuttra)	Alnylam	TTR/ hATTR-polyneuropathy	GalNAc-siRNA	2022	[10,38]
Nedosiran (Rivfloza)	Novo Nordisk	LDHA/ Primary hyperoxaluria type 1	GalNAc-siRNA (GalXC™)	2023	[13,66]
Fitusiran (Qfitlia)	Sanofi	Antithrombin/ Hemophilia A and B	GalNAc-siRNA	2025	[67]
Plozasiran (Redemplo)	Arrowhead pharmaceuticals	APOC3/ Familial chylomicronemia syndrome	GalNAc-siRNA (TRiM™)	2025	[68]

TTR, transthyretin; hATTR, hereditary transthyretin-mediated amyloidosis; ALAS1, aminolevulinate synthase 1; HAO1, hydroxyacid oxidase 1; PCSK9, proprotein convertase subtilisin/kexin type 9; LDHA, lactate dehydrogenase A; APOC3, apolipoprotein C-III; GalNAc, N-acetylgalactosamine; siRNA, small interfering RNA.

## Data Availability

No new data were created or analyzed in this study. Data sharing is not applicable to this article.
